# Safety of positive gastrointestinal contrast media. Updated guidelines by the ESUR Contrast Media Safety Committee

**DOI:** 10.1007/s00330-026-12399-6

**Published:** 2026-03-12

**Authors:** Carmen Sebastià, Aart J. van der Molen, Francisco Vega, Olivier Clément, Carlo C. Quattrocchi, Marie-France Bellin, Michele Bertolotto, Torkel Brismar, Jean-Michel Correas, Katerina Deike, Ilona A. Dekkers, Remy W. F. Geenen, Gertraud Heinz, Andreas H. Mahnken, Carlo A. Mallio, Alexander Radbruch, Peter Reimer, Giles Roditi, Laura Romanini, Fulvio Stacul

**Affiliations:** 1 Department of Radiology, Hospital Clinic de Barcelona, Barcelona, Spain; 2 Department of Radiology, Leiden University Medical Center, Leiden, The Netherlands; 3 Department of Allergy, Hospital Universitario de la Princesa, Madrid, Spain; 4 Université de Paris, AP-HP, Hôpital Européen Georges Pompidou, DMU Imagina, Service de Radiologie, Paris, France; 5 Centre for Medical Sciences CISMed, University of Trento, Trento, Italy; 6 University Paris Saclay, AP-HP, University Hospital Bicêtre, Department of Radiology, BioMaps, Le Kremlin-Bicêtre, France; 7 Department of Radiology, University of Trieste, Ospedale di Cattinara, Trieste, Italy; 8 Department of Clinical Science, Intervention and Technology, Unit of Radiology, Karolinska Institute and Department of Radiology, Karolinska University Hospital in Huddinge, Stockholm, Sweden; 9 Université de Paris, AP-HP, Groupe Hospitalier Necker, DMU Imagina, Service de Radiologie, Paris, France; 10 Clinic for Diagnostic and Interventional Neuroradiology, University Clinic Bonn, and German Center for Neurodegenerative Diseases, DZNE, Bonn, Germany; 11 Athinoula A. Martinos Center for Biomedical Imaging, Massachusetts General Hospital, Charlestown, MA USA; 12 Department of Radiology, Amsterdam UMC, Amsterdam, The Netherlands; 13 Department of Radiology, Northwest Clinics, Alkmaar, The Netherlands; 14 Department of Radiology, Landesklinikum St Pölten, St Pölten, Austria; 15 Department of Diagnostic and Interventional Radiology, Marburg University Hospital, Marburg, Germany; 16 Fondazione Policlinico and Research Unit Radiology, Universitario Campus Bio-Medico, Roma, Italy; 17 Department of Radiology, Institute for Diagnostic and Interventional Radiology, Klinikum Karlsruhe, Karlsruhe, Germany; 18 Department of Radiology, Glasgow Royal Infirmary, Glasgow, UK; 19 Department of Radiology, ASST Cremona, Cremona, Italy; 20 Department of Radiology, Ospedale Maggiore, Trieste, Italy

**Keywords:** Contrast media, Barium sulphate, Tomography (X-ray computed), Fluoroscopy, Adverse effects

## Abstract

**Abstract:**

Many reports on adverse effects related to positive gastrointestinal (GI) contrast media (CM) predate 2000; therefore, a literature review on their current safety profile was warranted. This article reviews the literature and updates the Contrast Media Safety Committee guidelines of the European Society of Urogenital Radiology on the safety of positive GI iodine-based and barium sulphate-based CM. A systematic literature search (2000–2025) identified 2 randomised controlled trials, 2 comparative studies, 17 reviews, and 29 case reports on the adverse effects of positive GI CM. Enteric non-ionic iodine-based low- and iso-osmolar CM are more palatable than ionic hyperosmolar agents (HOCM) and are preferred for oral use. The most frequent adverse effects of enteric ionic iodine-based HOCM are nausea, diarrhoea, vomiting, abdominal pain, and unpleasant taste, while pulmonary complications following aspiration are extremely rare. Hypersensitivity reactions due to limited (1–2%) systemic absorption of iodine-based CM are very uncommon; however, patients with a history of such reactions should be managed as for intravascular iodine-based CM administration. For barium sulphate CM, nausea, vomiting, and constipation are the most reported adverse effects. Minor leakage into the mediastinum or aspiration of small amounts into the lungs is rarely life-threatening. In contrast, intraperitoneal leakage can trigger inflammatory reactions, granuloma formation, and intestinal adhesions. If bowel perforation is suspected, fluoroscopic examination with iodine-based CM should precede barium administration. Hypersensitivity may occur due to excipients within barium preparations rather than barium itself.

**Key Points:**

***Question*** *What are the current safety issues associated with positive GI radiological CM*?

***Finding*** *Nausea and vomiting are the most commonly reported adverse effects of positive enteric CM. Many reports of other adverse effects date back to before 2000*.

***Clinical relevance*** *The use of positive GI CM has diminished in recent decades. However, these CM have excellent safety profiles and are safer than traditionally assumed*.

## Introduction

Gastrointestinal (GI) contrast media (CM) can be broadly classified as positive and neutral. Positive GI CM appear radiopaque on radiological tests, while the appearance of neutral CM, also described as negative or dark, resembles examples of which include water or gas [1].

Two broad categories of positive CM are the most commonly used: those based on iodine and those based on barium sulphate [1]. Barium sulphate-based CM has a long history of use in diagnostic imaging, having been first described in 1910 [2]. In addition, the earliest non-vascular use of iodine-based contrast media (ICM) was by oral administration in 1959 [3]. Many reports of the adverse effects related to positive GI CM date back before 2000, and thus, a review of the current literature on the safety of these CM was considered necessary [4].

Currently, the use of oral and rectal contrast administration in fluoroscopy and abdominal CT studies for the diagnosis of GI diseases has decreased dramatically. The indications for the use of CM for fluoroscopic examinations of the GI tract and judicious use of oral contrast in abdominopelvic CT were recently updated [1, 5–7].

GI CM for magnetic resonance imaging (MRI) are mainly neutral. In 2016, the first joint European Society of Gastrointestinal and Abdominal Radiology/European Society of Pediatric Radiology consensus statement on the technical performance of cross-sectional small bowel and colonic imaging led to the development of guidelines describing a standardised approach to patient preparation and acquisition protocols for MRI and CT of the small bowel and colon [8]. The current gadolinium-based contrast agents for intravenous use are not officially registered for GI use, and non-vascular use of these is considered off-label [9, 10].

The aim of this article is to review the literature and update the Contrast Media Safety Committee (CMSC) of the European Society of Urogenital Radiology (ESUR) guidelines on the safety of ICM and barium sulphate administered by a GI route [11].

## Materials and methods

A systematic search in PubMed was performed in search of studies on the adverse effects of barium sulphate-based and iodine-based GI CM. English literature worldwide was searched from January 2000 to September ﻿2025. The key terms searched included barium sulphate, meglumine, Gastrografin, diatrizoate meglumine, sodium diatrizoate, ioxitalamate meglumine, fluoroscopy, computed tomography (CT), oral water-soluble contrast, safety and complication. The search strategy involved the use of a combination of key terms and their variations. Based on title and abstract, one reviewer (C.S.), a radiologist with 24 years of experience in abdominal radiology, selected the articles. The inclusion criteria for eligible articles included clinical trials, randomised controlled trials, systematic/non-systematic reviews, and case reports on the safety and adverse effects of positive GI CM. The exclusion criteria encompassed studies not published in English, those using positive GI CT for purposes other than safety concerns, and studies that did not specifically utilise these contrasts. This strategy resulted in 2 randomised control trials, 2 comparative studies, 17 reviews and 29 case reports. The concept guideline was discussed by the CMSC members and consultants and approved at the CMSC Meeting in Lisbon (Portugal) in September 2024.

## Iodine-based GI contrast media

ICM are divided into ionic high osmolar and non-ionic low and iso-osmolar agents. Two ionic iodine-based high osmolar contrast media (HOCM)—diatrizoate meglumine/sodium and ioxitalamate meglumine—are commonly used for GI opacification. Different concentrations of non-ionic low-osmolar (low osmolar contrast medium/media (LOCM)) and iso-osmolar iodine-based contrast media (IOCM) are also approved for oral use in fluoroscopy and CT, as well as for rectal use in children. These CM are shown in Table 1.

**Table 1:** Positive GI contrast media

Group	Molecule	Chemical structure	Osmolarity	Water soluble
IODINE-BASED CONTRAST MEDIA				
Ionic iodine-based contrast media	Diatrizoate meglumine/sodium	Dissociable salt containing the meglumine cation	High-osmolar	Yes
	Ioxitalamate meglumine	Dissociable salt containing the meglumine cation	High-osmolar	Yes
Nonionic iodine-based contrast media	Iohexol^*^	Contains classic carbamoyl sidechains	Low-osmolar	Yes
	Ioversol	Contains classic carbamoyl sidechains	Low-osmolar	Yes
	Iodixanol	Contains classic carbamoyl sidechains	Iso-osmolar	Yes
	Iobitridol	Contains methylated carbamoyl sidechains	Low-osmolar	Yes
	Iopromide	Contains classic carbamoyl and a methylated carbamoyl sidechain	Low-osmolar	Yes
	Iopamidol^*^	Contains propane-based carbamoyl sidechains	Low-osmolar	Yes
BARIUM SULPHATE				
Barium sulphate	Barium	Crystalline solid inorganic compound		No

^*^Also available as a dedicated GI formulation, i.e. pre-diluted oral solution in selected markets (UK, USA)

ICM are generally the preferred agent for opacification of the GI tract in CT studies. For fluoroscopy, non-diluted ICMs are typically reserved for postoperative patients or patients with suspected bowel perforation where barium is contraindicated [4–7]. Notably, after dilution with water, all positive GI ICM used for CT are hypo-osmolar to plasma [1].

Non-diluted diatrizoate meglumine/sodium has transitioned from being a diagnostic contrast medium (CM) to a prognostic tool for distinguishing bowel obstructions that require conservative versus surgical management [12–15]. Diatrizoate meglumine/sodium is considered a therapeutic agent in cases of faecal impaction in children and in the constipated elderly with rectal administration, although a recent meta-analysis shows better therapeutic effect with oral administration [16, 17]. It was previously thought that enteral administration of non-diluted ionic HOCM had a therapeutic effect on intestinal obstruction, but this is now being questioned [18]. The theoretical mechanism of the therapeutic effect of diatrizoate meglumine/sodium is the hyperosmolarity of this agent. draws fluid into the bowel lumen [13, 14]. However, in children and frail elderly patients, this may lead to hypovolemia and dehydration, and thus, non-diluted ionic HOCM should be avoided in patients with fluid and/or electrolyte imbalances [18].

In recent literature, nausea, diarrhoea, vomiting, and abdominal pain are the most frequently reported adverse effects of GI ICM [19]. Enteric, non-ionic, iodine-based LOCM and IOCM are more palatable than ionic iodine-based HOCM, and their use as oral contrast is preferred [20, 21].

The greatest safety concern with ionic HOCM, particularly in swallowing tests or esophagography, is the potential risk of aspiration into the lungs, causing life-threatening pulmonary oedema. Almost all the literature published on this topic was published prior to 2000 [22–24]. On reviewing more recent literature regarding diatrizoate meglumine/sodium and pulmonary aspiration and/or pulmonary oedema, only three studies investigating their pulmonary toxicity in animals have been published [25–27]. Aspiration leading to pulmonary oedema has not been reported in articles about swallowing tests or esophagography with oral contrast (even with sedation) [28]. In a meta-analysis on the use of diatrizoate meglumine/sodium to reduce surgery in postoperative small bowel obstruction, “no Gastrografin-related complications (e.g., fluid or electrolyte disturbance, aspiration pneumonia, or exacerbation of obstructive episodes) were reported” [14].

The multiple adverse effects of ionic HOCM administered intravascularly cannot be extrapolated to oral or rectal administration of this CM. Only two case reports on iodine-induced hyperthyroidism and acute kidney injury following enteric administration of ionic HOCM have been published in the recent literature [29, 30].

GI administration of ICM results in 1–2% systemic absorption that could increase somewhat in patients with mucosal inflammation and bowel stasis [31]. Even minimal systemic absorption can lead to hypersensitivity reactions (HR), which are not dose-related. However, HR after nonvascular administration of ICM are very rare [31, 32]. The use of premedication to preclude HR is currently not considered useful, and thus, switching to an alternative CM based on the results of an allergy assessment, is preferable [32]. ESUR guidelines recommend that physicians take the same precautions for nonvascular as for intravascular ICM administration [11, 32, 33]. The risks and management of HR associated with the administration of ICM are discussed in the recent CMSC guidelines [34, 35].

## Barium sulphate

Barium sulphate is a solid, crystalline, inorganic compound that has no pharmacological activity and is neither absorbed nor metabolised by the body. It is excreted unchanged in the faeces, see Table 1. Barium sulphate suspension remains the CM of choice for fluoroscopic imaging, especially in swallowing studies. When a leak is suspected, ICM are preferred, even though 25% of leaks can be missed due to lower radio-opacity than barium [5].

In routine practice, oral barium CM has relatively few adverse effects. Patients most commonly complain of nausea and vomiting within 30 min of ingestion [36, 37]. Additional considerations include patients with constipation that predisposes the formation of barium impaction [5, 37]. Direct toxicity from accidental systemic absorption of barium is very rare but can result in changes in electrolyte balance, causing rapid and severe hypokalemia [12, 36].

Aspiration of barium sulphate suspension after oral administration is a rare, albeit well-recognised, complication of upper GI CM examinations [37]. In a review of cases with aspiration of barium sulphate suspension following esophagograms published from 1980 to 2018, only 28 cases of barium aspiration were identified, 20 of which were described after 2000 [37]. Since this latter review, only two more case reports of barium aspiration with clinical symptoms have been published [38, 39]. The severity of airflow obstruction and respiratory complications depends on the amount of barium sulphate suspension aspirated. Low-volume aspiration of barium sulphate suspension, frequently observed during swallowing studies, does not lead to any clinical sequelae. However, high-volume aspiration may lead to respiratory failure and circulatory shock with a high mortality rate [36–39]. Patients undergoing upper GI studies should be carefully selected. If oropharyngeal dysphagia is suspected, the ability of the patient to swallow without symptoms, such as coughing, should be assessed before the study is performed.

The most serious complication usually reported following the administration of barium sulphate suspensions in the GI tract is leakage into the peritoneal cavity [40]. Gastric, duodenal, small intestinal, and colonic barium sulphate suspension leakage may result in barium peritonitis and residual barium sulphate in the intraperitoneal cavity can cause persistent inflammatory reactions [40]. Historically, leakage of barium sulphate from the colon carries the highest mortality, likely related to infection from leakage of stool and risk of subsequent sepsis [40–42].

On review of the literature on barium sulphate-induced peritonitis from 2000 onwards, only 10 case reports of leakage of barium into the peritoneal cavity leading to relevant associated symptoms have been published [42–52]. The presence of barium in the peritoneum can cause a severe inflammatory reaction, the treatment of which involves surgical lavage with a large volume of normal saline. If surgical treatment is not performed, leaked barium particles may remain in the mesentery and peritoneal surface and may compromise follow-up studies indefinitely [36]. Residual barium in the intraperitoneal or retroperitoneal cavity can be a persistent source of inflammation, leading to granuloma formation and intestinal adhesions [36, 42–52]. Nowadays, the use of corticosteroids or surgery in cases of leakage of barium sulphate in the peritoneal cavity has no evidence of utility [40].

The toxicity of barium sulphate suspensions to mediastinal tissues is doubtful. While it has been experimentally shown that leakage of barium sulphate suspensions into the mediastinum may cause an inflammatory reaction with subsequent granuloma formation and fibrosis, there is little to no evidence that barium sulphate causes clinically significant mediastinitis [5]. Oesophagograms may be performed safely with diluted barium to rule out postoperative anastomotic oesophageal leaks, with no cases of mediastinitis having been described, although the current state-of-the-art is to perform a CT study [53]. In a series of barium leakages during oesophagography, no adverse effects from barium leak in the mediastinum were reported [54].

In the past, the frequency of HR after the administration of barium sulphate suspensions has been reported to be 1 in 750,000 examinations, with most manifestations being mild [55]. Barium sulphate is not considered a substance capable of inducing HR. Cases of HR following the administration of barium sulphate suspensions are likely due to patient sensitisation to carboxymethylcellulose [56] or carrageenan [57], which are used as excipients in the commercial formulations of barium sulphate, or to latex present in the administration devices used for rectal enemas [58]. Therefore, patients with any of these three hypersensitivity conditions are at increased risk of a HR after a radiological examination with barium sulphate suspensions. If there is any doubt, an allergologic evaluation with the CM should be performed before the examination. In cases of latex allergy, latex-free administration devices should be used.

## Conclusion

The scarcity of reports of adverse effects of positive oral and rectal GI CM in the literature after 2000 could be the result of a more appropriate use in at-risk patients, the more frequent use of non-ionic LOCM, fewer published case reports of adverse reactions, studies that are more focused on diagnosis and not on the adverse effects of CM, and the reduction of indications for GI contrast media in daily practice.

The classical adverse effects of GI CM, both ionic high-osmolar ICM and barium sulphate suspensions (aspiration and mediastinitis), date back to articles published before 2000. Aside from a few case-reports, these adverse effects have been scarcely reported after 2000. Thus, the lack of recent publications on the adverse effects of GI CM makes it very difficult to perform a reliable systematic review of the recent literature and does not allow robust evidence-based recommendations to be made. To guide clinical practice in the use of positive GI contrast media, the ESUR CMSC has formulated a number of expert-based statements and recommendations (Table 2).

**Table 2:** ESUR CMSC statements and recommendations

Iodine-based contrast media
- Nausea, diarrhoea, vomiting and abdominal pain are the most reported adverse effects of enteric iodine-based CM.
- Non-ionic iodine-based LOCM and IOCM are more palatable than ionic HOCM, and their use as oral contrast is preferred.
- Enteric ionic iodine-based HOCM should be avoided in patients with fluid and/or electrolyte imbalances; if possible, use non-ionic LOCM or IOCM.
- Pulmonary complications after aspiration of enteric ionic iodine-based HOCM into the lungs are extremely rare.
- Side effects reported after intravenous administration of ICM cannot be extrapolated to gastrointestinal administration; the frequency of occurrence is much lower for oral /rectal administration.
- HRs due to 1–2% systemic absorption following enteric administration are very rare.
- Patients with a history of HRs to iodinated CM should be managed similarly to management following intravascular iodinated CM administration.
**Barium sulphate-based contrast media**
- Nausea, vomiting and constipation are the most reported adverse effects of barium sulphate-based CM.
- Constipation predisposes to the formation of barium impaction.
- If bowel perforation is suspected, begin the fluoroscopic exam with iodinated CM prior to administering barium.
- Leakage of barium into the peritoneum can lead to an inflammatory reaction, granuloma formation and the development of intestinal adhesions.
- A small amount of pulmonary barium aspiration is relatively safe.
- There are no reported cases of mediastinitis due to leakage of barium into the mediastinum after 2000.
- Patients may experience hypersensitivity to excipients of barium formulations but not directly to barium.

*ICM* iodine-based contrast medium, *CM* contrast media, *HOCM* high-osmolar contrast medium, *LOCM* low-osmolar contrast medium, *IOCM* iso-osmolar contrast medium

Future research on positive oral radiological contrast media should focus on evaluating their role in modern imaging protocols, particularly in comparison with neutral agents, to determine their impact on diagnostic accuracy and patient outcomes. Studies are needed to assess the safety and tolerability of existing formulations in contemporary practice, as well as to explore the development of new agents with improved profiles and fewer adverse effects. Additionally, research should investigate their potential applications in emerging technologies such as dual-energy CT and AI-assisted image analysis, ensuring that clinical guidelines reflect current evidence and optimise patient care.

## Abbreviations

CM: Contrast medium/media
CMSC: Contrast Media Safety Committee
CT: Computed tomography
ESUR: European Society of Urogenital Radiology
GI: Gastrointestinal
HOCM: High osmolar contrast medium/media
HR: Hypersensitivity reaction/reactions
ICM: Iodine-based contrast medium/media
IOCM: Iso-osmolar contrast medium/media
LOCM: Low osmolar contrast medium/media
MRI: Magnetic resonance imaging
